# Visual Quality Assessment after Network Transmission Incorporating NS2 and Evalvid

**DOI:** 10.1155/2014/267403

**Published:** 2014-04-15

**Authors:** Zhengyou Wang, Wan Wang, Yanhui Xia, Zheng Wan, Jin Wang, Liying Li, Cong Cai

**Affiliations:** ^1^Shijiazhuang Tiedao University, School of Information Science and Technology, Shijiazhuang 050043, China; ^2^Jiangxi University of Finance & Economics, School of Information Technology, Nanchang 330032, China; ^3^Shijiazhuang Tiedao University, School of Economics and Management, Shijiazhuang 050043, China

## Abstract

On the basis of Evalvid tool integrated in NS2 (Network Simulator version 2), the paper gets new set of tools, myEvalvid, to establish the simulation and evaluation platform for multimedia transmission. Then the paper investigates the effects of various influence factors when multimedia information is transmitted in the network and the relationships among these factors. Based on the analysis, the paper gets different evaluation models, respectively. In this paper, we study the impact on performance of several basic source and network parameters of video streams, namely, GOP (Group of Pictures) pattern, compression quantitative parameters, packet length, and packet error rate. Simulation results show that different parameters lead to different distortion levels which are calculated according to the reconstruction images at the receiver and the original images. The experimental results show that the video transmission and quality evaluation model we designed can evaluate multimedia transmission performance over complex environment very well.

## 1. Introduction 

Image quality evaluation is one of the most important steps in the field of image processing. In all aspects of image processing, such as image compression, image transmission, and image deblur, image quality evaluation plays a very important role. Image quality evaluation methods can be divided into subjective evaluation method and objective evaluation method according to the subject of evaluation. The researchers often use mean opinion score method and the peak signal-to-noise ratio method. Over the past years, a lot of researches have been conducted towards the construction of objective video quality metrics. Applying NS2 (Network Simulator version 2) and Evalvid, video streams could be transmitted through the simulation network and the corresponding peak signal-to-noise ratio (PNSR) could be calculated. And the image transmission quality in different network environments, the influence factors on the quality of the image transmission can be analized of image transmission quality in different network environments and the influence factors on the quality of the image transmission.

Recently, more and more telecommunication systems support different types of real-time transmission, and video transmission is one of the most important applications. The growing needs lead to the support of the video quality evaluation. Although many papers have been committed to study the QoS (Quality of Service) mechanism which is supported through different types networks, many researchers are limited to the ranges of using packet loss rate, packet delay, and packet jitter as video transmission quality metrics. As known to us all, the above metrics cannot be easily transformed into video transmission quality. In fact, this relation may be due to coding scheme, hide loss scheme, and delay or jitter processing which are not the same leading to differences. To make detailed descriptions to these related parameters, we use NS2 and myEvalvid tools as simulation platform to obtain the transmission results. Obtained correlations among these parameters are of great significance to the evaluation of video quality.

The paper creates the platform to simulate and evaluate the transmission image in the multimedia network. Various factors that affect the quality of video transmission are probed and the correlations among these factors are achieved. As for the issue of the simulation of video stream, the authors apply the method of introducing the traffic trace file of video stream to the network simulation circumstance, aiming to simulate the actual transmission of video stream in the network. Thus, the author can get the record file sd (send data) of sender and the record file rd (receive data) of receiver and use them to make an effect assessment. Different effects caused by various factors during video transmission can be also evaluated. Evaluation model could be established on the basis of these experimental findings. The results reveal that various value settings of GOP (Group of Pictures) pattern, quantization value, packet size, and packet error rate lead to different distortions between the original video and the reconstructed video.

The remainder of this paper is organized as follows.  Section 2 discusses related work of image quality assessment. The experimental environment is described in detail in Section 3 and experimental PSNR values are obtained in Section 4. In Section 5 we build linear regression model of based network parameters and analyze and explain experimental results. And a general conclusion is presented in Section 6.

## 2. Related Work

Reference [1] used ANOVA (Analysis of Variance) to analyze the delay, jitter, packet loss, and their relationships in the video quality assessment and safely draw a conclusion that jitter and packet loss have significant influences on video quality. Furthermore, the influence degree of the jitter and packet loss is equal to the video quality. Reference [2] puts forward all the multimedia signal processing algorithm need appropriate Fidelity index verification results. It makes use of the human visual system (HVS)-image processing based on objective fidelity metrics. And this method is described for a set of special image which is called region of interest (ROI) of the measurement problem. Moreover, this paper has not built video quality model. ITU-TG-1070 (International Telecommunication Union Telecommunication-G) [2] provides video quality evaluation model which takes packet loss as variable for video phone. Reference [3] studied the video quality evaluation under the situation of burst packet loss based on [2]. The improved model (T-Model) draws into the complement factor B and comes to describe the burst degree of continuous packet loss. References [4, 5] aimed at using MPEG-2 (Moving Picture Experts Group) coding to research the relationship between the video quality evaluation and packet loss and the relationship between the video quality evaluation and coding rate. It takes the method of MPQM (Moving Pictures Quality Metric). Grading the video quality finally gets the grading value Q of the video quality. These video quality evaluation models only considered the influences of packet loss, while they do not consider the influences of other factors such as QoS factors (delay and jitter). In [6] the author presented a metric based multichannel model of human spatiotemporal vision that has been parameterized for video coding applications. Evaluation method of video evaluation QoE (Quality of Experience) can be divided into subjective evaluation method and objective evaluation method. Subjective evaluation methods are Mean Opinion Score (MOS), Distortion Mean Opinion Score (DMOS) [7], Single stimulate continuous Quality Score (SSCQS) [8], Double Stimulate Continuous Quality Score (DSCQS) [8], and so on. Subjective evaluation method's advantages are accurate and easy to understand, but it is hard to implement because steps are complex and it is hard to implement in the laboratory environment. Objective evaluation methods often use PSNR, MDI (Media Delivery Index) [9], NTIA (National Telecommunications and Information Administration) [10] model, and so on. In PSNR method, PSNR values are obtained through comparing source files and those after decoding pixel-by-pixel, the results can not accurately reflect the quality of the user's experience [11]. MDI consists of two parts: delay parameters and media loss rate. This evaluation method's premise is “if the transmission quality is good, then the video quality is good.” MDI directly uses network parameters to show video quality because MDI provides no quantitative score evaluating network video quality. Thus the video quality researches rarely use this method. ITU-TJ.144 (International Telecommunication Union Telecommunication-J) compares and analyzes the different objective evaluation and subjective evaluation models fitting degree in [12], and the results show that comprehensive performance of NTIA model is the optimal in all chosen models (the Pearson correlation coefficient is 0.938; the mean square error is only 0.074). Furthermore, this study also used this model to evaluate the video after transmission. This model's algorithm has already been implemented in the video quality evaluation software BVQM (Batch Video Quality Metric) which was developed by telecommunications academy of sciences ITS (Institute for Telecommunication Sciences). Reference [13] proposed a content-adaptive packet-layer (CAPL) model for networked video quality assessment, by evaluating the distortions induced by both compression and packet loss. This paper took the regression model to get the mapping relationship between the QoS index and QoE. Reference [14] proposed a kind of network QoS index affecting Experience Quality (QoE) estimation based on WiMAX (Worldwide Interoperability for Microwave Access). This method is more accurate than the previous. Reference [15] illustrated the influences of the packet loss on the quality of video, and reference [13] put forward a kind of content adaptive model, considering video complexity in time domain and airspace views. Based on the above references, we can extract network parameters from kinds of aspects for our research.

## 3. Experiment Environment

Since in the real network environment the parameters of the network cannot be set to realize controllability and repeatability of the network damage, the paper presents the design of the simulation platform to simulate the process when the users watch video. The network transmission module is used to set up different QoS parameters and control network damage. Video quality evaluation module is used to compare the source video files and target video files to get user experience quality.

In order to improve Evalvid and to enhance the function of simulation, we can make Evalvid through the myEvalvid, my_UDP, and myEvalvid_Sink, which are three interface programs (or we can say agent) communicated with NS2. The integration is myEvalvid [20]. Below we aim at giving a simple introduction to myEvalvid, my_UDP, and myEvalvid_Sink.

1. myEvalvid: the interface program's main job is to learn to read VS (Video Sender) procedures from the film log files. The log files of each picture are cut into smaller segments, and the user in Tcl (Tool Command Language) Script can set in good time to the section which is the bottom of the UDP layer sending out.
2. my_UDP: basically my_UDP agent is the extension of UDP agent. The new agent has the packet transmission time, packet identification, and packet load size recorded in the files which are set up by Tcl Script. Simply say, generally My_UDP's work is as Tcp-dump or Win-dump's work.
3. myEvalvid_Sink: it is responsible for receiving packets which are issued by my_UDP. This agent records the receive time, packet identification, and packet load size in the files which are specified in Tcl Script.

## 4. Image Transmission Effect Analysis and Evaluation

### 4.1. Use MyEvalvid for PSNR Evaluation

**Figure 1:**
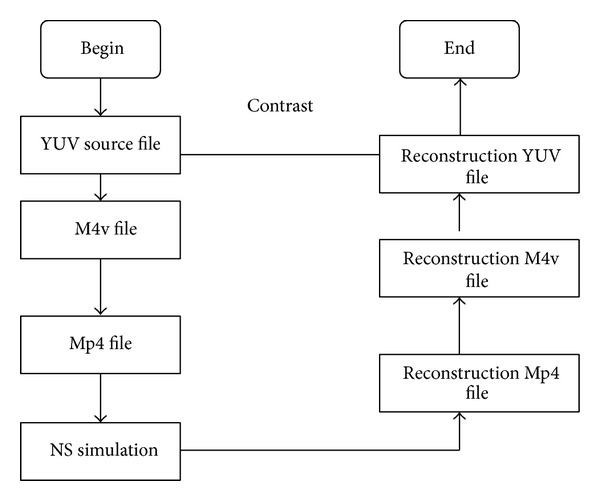
Image transmission quality evaluation process.

Using myEvalvid to evaluate image transmission quality, Figure 1 shows the process of implementation. We firstly use the source YUV file to generate NS2 simulation file. After the NS2 network simulation, we reconstruct YUV file at the receiving end. By comparison and analysis, we can calculate the value of PSNR and evaluate the quality of video transmission.

**Figure 2:**
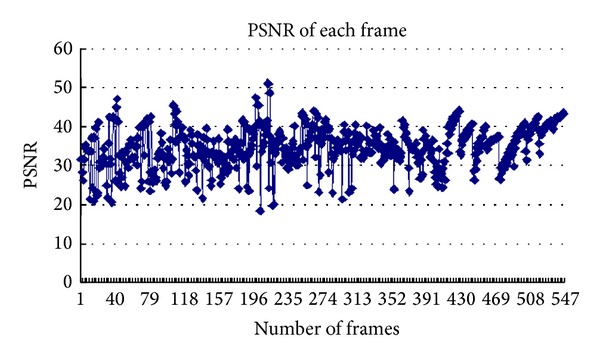
Each frame PSNR value of akiyo video stream.

This paper uses akiyo_cif as an example and calculates PSNR value of each frame. A plot curve of results is shown in Figure 2. Through the network transmission, the image's average PSNR value is 36.92.

We can apply YUV viewer software to observe video frame distortion situation.

**Figure 3:**
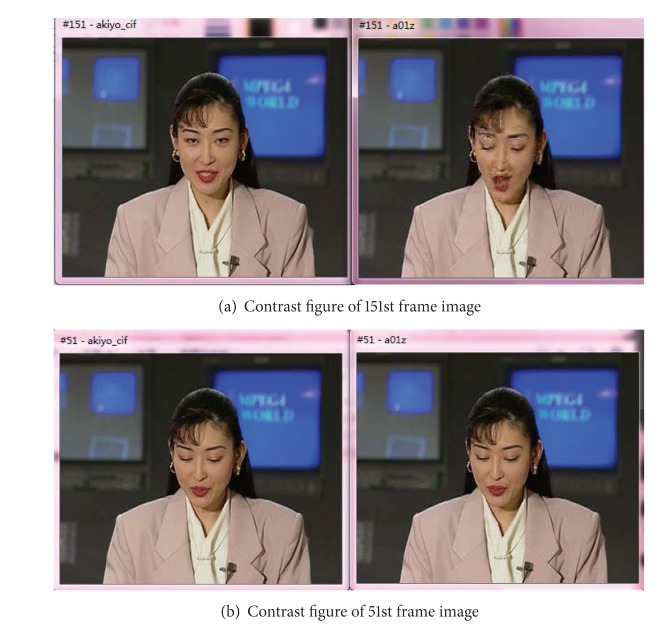
Contrast figure of original image and reconstruction image.

When using myEvalvid to verify the multimedia network structure, we can calculate the PSNR of reconstructed video, which can be used to observe the difference between the received movie and the original movie. Taking 151st frame as example, the received PSNR value is 27.24. Compared with the 51st frame, the received PSNR value is 45.48, as shown in Figure 3.

As shown from Figure 3, the image on the left side is better than the image on the right side, owing to simulation transmission process in the above network. During packet transmission process, there will be some packet loss. Thus it will lead to some pictures becoming in unsolvable state. Therefore after arriving at the receiving end, the reconstructed image quality is worse than the original image.

In network simulation transmission process, there are packet losses in the network. Since packet loss probabilities of different pictures are distinct, some pictures are in unsolvable state and the others are in solvable state. Therefore, distortion level of individual frame of the video stream will vary. That is to say, packet loss rate has a great influence on the quality of image transmission.

## 5. Parameters' Influence of Image Transmission 

### 5.1. Influence Factors of Image Transmission

For different multimedia streams, the quality of the transmission is distinct because of compression parameters, network parameters, and network states. In the network transmission, possible multimedia factors generally include the following: GOP pattern, quantization value, packet size, and packet error rate.

### 5.2. Parameters' Influence on the Quality of Image Transmission

Through the above analysis, we can change the settings of the parameters and conduct simulation experiment to obtain data. From the data analysis, we can discuss the influences of various kinds of factors on the quality of image transmission.

(1) The influences of quantization parameters on the quality of image transmission; quantization is a process that attempts to determine which information can be safely discarded without a significant loss in visual fidelity.

When the quantization parameters are set to 31, 20, and 10, the PSNR values of simulation results are shown in Table 1.

From Table 1 we can know that when performing compression, the larger the value of the quantization parameter is, the worse the image quality is. This is because, in the image compression process, when the setting of quantitative parameters is bigger, the image quality after compression will be worse. Therefore, the image quality at the receiving end after the network transmission is worse than that with a small quantitative parameters setting. When using smaller quantitative parameters, it will have a good image effect. At the same time, we can also find that the more quantization parameters the data compressed, the more necessary it is to transmit packets.

(2) The influences of packet length in the quality of image transmission: to explore the influences of packet length in the quality of image transmission, we can change the packet size and MTU (Maximum Transmission Unit) values and keep other parameters constant. After conducting simulation, we can get different packet sizes and their correspondent average PSNR values. The processes of the experiments are complied with compression quantitative parameters simulation in the quality of the same image transmission. The results are shown in Table 2.

From Table 2 we can find that, when the packet length is longer, the image quality will be higher. The reason of this phenomenon is, for the same image, if the packet length is long, on behalf of each picture it is required to split the packet number less. Since packet error rate is the same, compared to shorter packet length, the lost packet number will be less. In this case more pictures could be decoded, leading to higher image quality. When using shorter packet length, on behalf of each picture it is required to split the number of packets more, so the lost packet numbers will also be more. In this case, relatively few pictures can be decoded, resulting in poor image quality.

(3) The influences of GOP pattern in the quality of image transmission: to explore the influences of GOP pattern in the quality of image transmission, we can set parameters of the GOP and calculate PSNR value to get the connection between the GOP and the quality of image transmission. The simulation results are shown in Table 3.

We can find from Table 3 that the shorter the image GOP length is, the better its quality is. This is because I frame packet loss will lead to pictures of the same GOP becoming noncoding. So in the image with longer GOP length, I frame loss has to wait for a long time to wait the next I frame arrival. As for the image with shorter GOP length, it will be waiting for the next I frame whose coming time is short. So the recovery time is shorter, resulting in relatively good image quality.

### 5.3. Network Video Mapping Model of QoE and QoS

#### 5.3.1. Establishing Evaluation Model

The two examples cited above are akiyo_cif and foreman_qcif because the two videos have different characteristics, considering the node, video dynamic, action, and complexity. Thus we make observation and comparison on different behaviors of each video and get the final results. It can be divided into the following steps to complete the evaluation model.

First of all, conduction video is streaming under the reservation condition, with Evalvid frame integrated into NS2 module. Each of the two selected videos is simulated fifty times. All of the schemes, namely, 1, 2, 3, and 4 nodes, include a total of 400 times transmission.

In the second step, we perform the statistical analysis. The goal is to get a more reliable and consistent database and eliminate the problems which may occur. Therefore, those who are away from the standard deviation value will be eliminated. one of these records contains the following QoS parameters: delay, jitter, and packet loss. This loss is calculated into total loss of a video. The loss refers to each frame's MPEG codec (I, P, and B).

In the third step, the original and decoded video file sequence are used to obtain QoS and QoE index. Using myEvalvid can obtain QoS index (jitter, delay, throughput, and packet loss), MOS, and PSNR but cannot obtain more stable index, such as structural similarity and VQM (Video quality Metric). However, we can combine packets which are transmitted through myEvalvid to get QoS index.

The last step is to multiple nonlinear regression equation to establish evaluation model and to evaluate its performance in the training sets and testing sets.

Finally, through fitting, we get the superiority of the evaluation model.

#### 5.3.2. Packet Loss and Jitter Influence on the Video Quality

It can be seen from the front of the research that delay has nothing to do with video's user quality of experience, while the jitter and packet loss have great effects on the video's user quality of experience. Thus the research of network effect of multiple factors on video transmission draws the conclusion that it is mainly the combined effect of jitter and packet loss on the video of the user quality of experience. On the other hand, in the actual network conditions jitter and packet loss exist at the same time and have constant changes. So it is of more practical significance to research the jitter and packet loss's comprehensive influence on video's user quality of experience. It can be concluded that the influence of packet loss and jitter on the video quality is nonlinear relationship, so this paper adopts multiple nonlinear regression equation to establish nonparametric model. The regression equations are as follows:

(1) $$\begin{array}{c} \text{MQ}=\alpha_{1}*\text{DP}+\alpha_{2}*\text{JT}+\mu_{1}, \end{array}$$

(2) $$\begin{array}{c} \text{VQ}=\beta_{1}*\text{DP}+\beta_{2}*\text{JT}+\mu_{2}, \end{array}$$

where MQ is mean quality, the average quality index of video sequences; VQ is varquality, the quality of video sequence variance; DP is droprate, packet loss rate; JT is jitter, Jitter.

Considering DP and JT separately, we can calculate their influence of MQ and VQ respectively.

(a) The influence of DP on MQ: regression model is shown in
  (3) $$\begin{array}{c} \hat{\text{MQ}}=11.06955-0.0058637*\text{DP}. \end{array}$$
(b) The influence of DP on VQ: regression model is shown in
  (4) $$\begin{array}{c} \hat{\text{VQ}}==18.15667-0.0356798*\text{DP}. \end{array}$$

From Tables 4 and 5 we can find that packet loss rate DP for video quality variance VQ has more explanatory power relative to the average video quality MQ.

(c) The influence of JT on MQ: regression model is shown in
  (5) $$\begin{array}{c} \hat{\text{MQ}}=10.68654-4.67\exp t\left(-6\right)*\text{JT}. \end{array}$$
(d) The influence of JT on VQ: regression model is shown in
  (6) $$\begin{array}{c} \hat{\text{VQ}}=15.85106-0.0000264*\text{DP}. \end{array}$$

From Tables 6 and 7, we can find that explanatory power of JT is not strong for MQ and VQ. But in the 5% level of significance, it is statistically significant. And if making a fusion of these two network parameters, MQ and VQ can get a good degree of interpretation and estimation.

**Figure 4:**
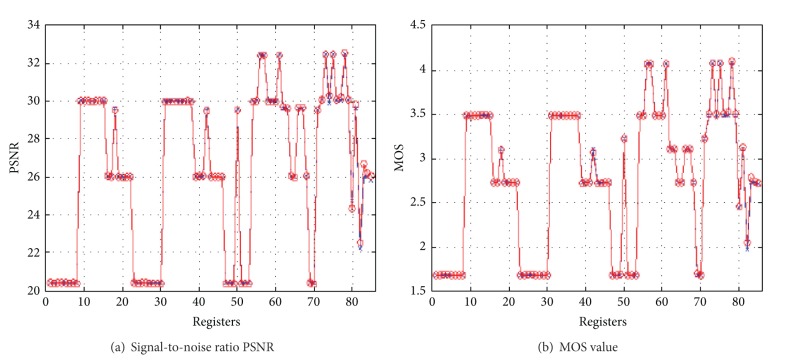
PSNR value and MOS value of first ninety frames.

Taking first ninety frames of akiyo streaming video as example, we can draw PSNR value and MOS value of line charts. In Figures 4(a) and 4(b), the abscissas represent the frames, and the ordinates represent the PSNR and MOS values.

As shown in Figure 4, when observing the same processing to the video and the characteristics of the complexity, we can find that MOS values are not significant. There is even more and more competition that it has influences on video broadcast node. For akiyo streaming video which has a node in the competition, the average score is about 3.6 and the standard deviation is 0.25. The average of four nodes is close to 3.4, and the standard deviation is 0.19. For video foreman that has a higher level of complexity and movement, the results are worse. As expected, each node of the MOS reduces. The study uses a large number of experimental results (include experimental results used for modeling and not used for modeling) and compares to the MOS value which is obtained by model calculation. The result error is no more than 0.4 and accuracy is within the acceptable ranges.

## 6. Conclusion

The paper studied the video transmission and quality evaluation issue over NS2 and Evalvid based simulation networks. Contributions of this paper are summarized in the following.

1. Verify the influences of all kinds of factors when investigating the quality of image transmission, by changing values of the parameters.
2. Establish effective evaluation model on the basis of influence analysis of different factors, using linear regression model.

In the simulation platform, we implement the transmission and quality evaluation of video streams. The results show that the video transmission and quality evaluation model we designed can evaluate the multimedia transmission performance over complex environment very well, and it could be a powerful tool in multimedia transmission research.

As for future work, we plan to study the impacts of network QoS on QoE of video, based on the model proposed in this paper. We will establish evaluation model of QoS to QoE and apply it to the network monitoring system which uses QoE as evaluation standard.

## Figures and Tables

**Table 1:** The average PSNR value of simulation results.

Compression quantitative parameters (*Q*)	Average PSNR
31	26.83
20	28.82
10	32.28

**Table 2:** The simulation results of the average PSNR value.

MTU	Average PSNR
1000	31.06
800	29.98
600	28.58
400	27.82

**Table 3:** The simulation results of the average PSNR value.

GOP	Average PSNR
9	32.28
15	32.09

**Table 4:** Statistical parameters of DP on MQ.

	*t*-statistics	*R*-square	Adjusted *R*-square	The 95% confidence interval
DP	−5.68	0.4021	0.3897	[−0.0079424, −0.0037906]

**Table 5:** Statistical parameters of DP on VQ.

	*t*-statistics	*R*-square	Adjusted *R*-square	The 95% confidence interval
DP	−6.73	0.4856	0.4749	[−0.0467363, −0.0252389]

**Table 6:** Statistical parameters of JT on MQ.

	*t*-statistics	*R*-square	Adjusted *R*-square	The 95% confidence interval
JT	−3.00	0.1578	0.1403	[−8.25*e* − 06, −1.63*e* − 06]

**Table 7:** Statistical parameters of JT on VQ.

	*t*-statistics	*R*-square	Adjusted *R*-square	The 95% confidence interval
JT	−3.52	0.2050	0.1885	[−0.0000494, −0.0000135]
